# Disparities in inpatient treatment and expenditures among lung cancer patients under tiered social health insurance: a population-based study in China

**DOI:** 10.1186/s12939-025-02533-z

**Published:** 2025-06-05

**Authors:** Yaoyun Zhang, Yu He, Qing Wang, Ying Meng, Xinxin Xia, Xiaokang Ji, Qingbo Zhao, Yongchao Wang, Yifu Zhao, Chao Lv, Liming Zhu, Ding Wang, Suping Ling, Fuzhong Xue, Jin Xu

**Affiliations:** 1 School of Public Health, Peking University, Beijing, China; 2 China Center for Health Development Studies, Peking University, Beijing, China; 3 Chinese Preventive Medicine Association, Beijing, China; 4 School of Public Health, Shandong University, Jinan, China; 5 Department of Thoracic Surgery II, Key Laboratory of Carcinogenesis and Translational Research (Ministry of Education/Beijing), Peking University Cancer Hospital & Institute, Beijing, China; 6 Zhejiang Cancer Hospital, Hangzhou, Zhejiang China; 7 National Administration of Health Data, Jinan, Shandong China; 8 London School of Hygiene & Tropical Medicine, London, UK; 9 World Health Organization Collaborating Center for Universal Health Coverage, Beijing, China

**Keywords:** Lung cancer, Disparity, Treatment, Expenditures, Social health insurance

## Abstract

**Introduction:**

Tiered social health insurance (SHI) schemes exist in many countries and may lead to significant disparities of healthcare and financial protection. The degree of cancer care inequalities under tiered SHI in China and other low- and middle-income countries (LMICs) remain poorly understood.

**Methods:**

We obtained hospital discharged summary for 319,677 patients diagnosed with lung cancer between 2017 and 2021 in Shandong, China, and established propensity score-matched samples under the Urban and Rural Resident Basic Medical Insurance (URRBMI) and those under the Urban Employee Basic Medical Insurance (UEBMI). We ran multivariable regressions to assess the effects of SHI schemes on cancer treatment and expenditures. Subgroup analyses of cancer treatment were conducted based on whether the cancer had metastasized.

**Results:**

In the matched samples, utilization of inpatient cancer care increased under both schemes from 2017 to 2021. Higher proportions of inpatient cancer care utilization were seen in those under UEBMI compared those under URRBMI, consistently with statistical significance. UEBMI was associated with a higher probability of receiving surgery in patients without metastasis, and higher probabilities of receiving radiotherapy or chemotherapy, targeted therapy, and immunotherapy in patients with metastasis. Patients under UEBMI were also less likely to be discharged against medical advice than those under URRBMI. Furthermore, UEBMI beneficiaries had 13.3% higher total expenditures but 19.1% lower out-of-pocket expenditures.

**Conclusions:**

Significant gaps remained in access to inpatient treatment and financial protection for lung cancer, particularly in surgery for non-metastatic cancer. Targeted harmonization of benefit packages is needed to address pressing disparities in cancer care in LMICs with tiered SHI.

**Supplementary Information:**

The online version contains supplementary material available at 10.1186/s12939-025-02533-z.

## Introduction

Risk pooling for health care, frequently established through social health insurance (SHI), is expected to enable patients to access essential health services when needed without financial hardship [1, 2]. Many countries are working on attaining universal health coverage (UHC) by implementing health insurance plans for their residents. However, unequal SHI coverage, particularly for diseases associated with high expenditures like cancer, could make substantial differences in both access to quality services and financial vulnerability [3, 4]. In health systems with tiered pools of SHI, which are common in low- and middle-income countries (LMICs) [5, 6], disparity of benefit package or copayment rates means potentially significant disparities of cancer care and financial protection [7–11].

In China, approximately 95% of the population was covered by two main types of SHI in 2023 [12]. The Urban Employee Basic Medical Insurance (UEBMI, established in 1998) funded through premiums contributed by employers and individuals covers employees and retirees in the formal sector, who constitute about 28% of all SHI beneficiaries, merged in 2016 from two other previously existing schemes that respectively covered urban and rural residents outside the formal employment sector [13]. The other main scheme is the Urban and Rural Resident Basic Medical Insurance (URRBMI) [13]. UEBMI and URRBMI share the same benefit package. However, the former is more generous reimbursement rates than the latter, though the gap in-between has been narrowing. In 2023, the average reimbursement rates for inpatient expenses under UEBMI and URRBMI were 84.6% and 68.1%, respectively [12]. Detailed background information on China’s SHI can be found in Part 1 of Supplemental Material. Understanding the disparities in cancer care between schemes may provide important insights for narrowing the benefit gaps in the context of tiered SHI [5, 14].

A literature review from the United States found that cancer patients with no or inferior insurance coverage had lower utilization of high-cost treatments and systemic treatments, and higher chances of treatment delays [15]. In the context of lung cancer, despite recent advancement in treatment technology for patients with this condition, international studies consistently demonstrated significant disparities in cancer care and economic burden across health insurance schemes [7–11, 16]. A small number of studies from China indicated that SHI coverage was associated with improved inpatient service utilization [9, 17]. One study found that low reimbursement rates of precursors of URRBMI schemes restricted access to tertiary facilities, relative to schemes with higher reimbursement rates [9]. Meanwhile, health insurance has been shown to protect some households from the impact of catastrophic health expenditure [18], though the overall effect was limited [19]. Few existing studies have explored the effects of tiered SHI on cancer care disparities, particularly in China and other LMICs.

Lung cancer is the leading cause of cancer incidence and mortality in China [20], with age-standardized incidence rates of 209.6 and 197.0 per 100,000 for males and females, respectively, and mortality rates of 127.5 and 67.8 per 100,000 in 2022 [21]. This situation imposes a substantial medical and economic burden on both patients and society [21, 22]. However, little is known about the inequalities regarding lung cancer care and expenditure caused by the tiered SHI in China. Enabled by a large real-world dataset of hospital discharge data from Shandong province (one of the most populous provinces in China with a reported lung cancer incidence rate of 327.52 per 100,000 in 2018 [23]), we sought to investigate the disparities of inpatient lung cancer treatment and related expenditures between the URRBMI and UEBMI beneficiaries.

## Materials and methods

### Data source and study population

Data were sourced from Shandong Province, a coastal province in eastern China, selected for its population of approximately 101.2 million, economic development comparable to that of an upper-middle-income country [24], and a notably high prevalence of lung cancer [23]. We used standardized hospital discharge data, formally known as hospitalization record front pages (HRFPs) from all secondary and tertiary hospitals in Shandong stored in the Cheeloo Lifespan Electronic Health Research Data-library (Cheeloo LEAD) (see Supplemental Material for details). HRFPs cover basic socio-demographics of patients, detailed information about disease diagnosis, treatment, and expenditure. Data on the platform were deidentified, with data for an individual linked via a unique encrypted identity number.

**Fig. 1:**
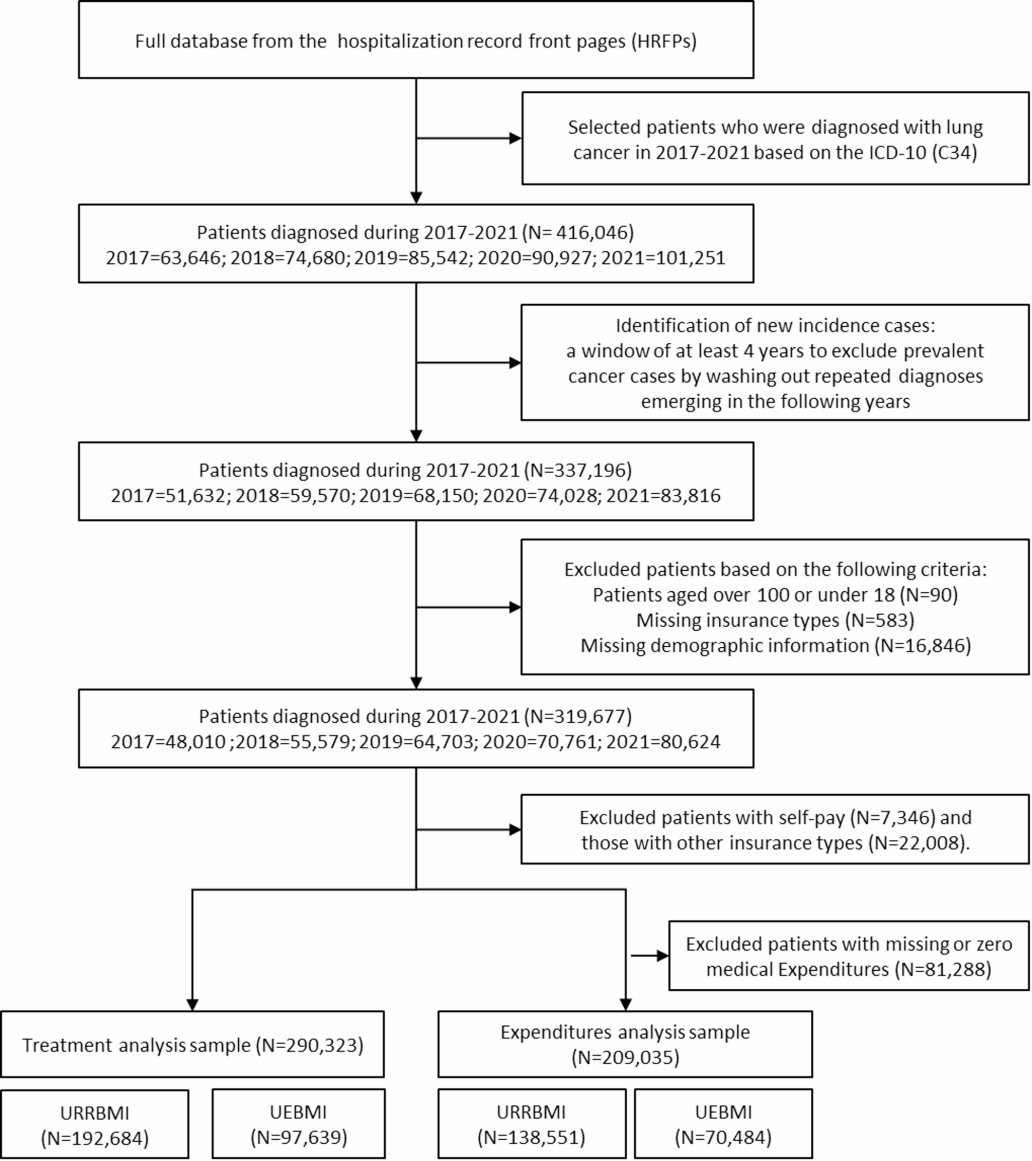
Sample derivation. *Note.* Abbreviations: UEBMI, Urban Employee Basic Medical Insurance; URRBMI, Urban and Rural Residents Basic Medical Insurance

We selected patients who were diagnosed with lung cancer (ICD-10: C34) and under URRBMI or UEBMI from January 1, 2017 to December 31, 2021. We set a window of at least four years to exclude patients with whom lung cancer had been previously diagnosed, by washing out repeated diagnoses emerging in the following years. The four-year wash-out period was adopted as the number of new cancer cases in 2021 remained stable when the time window was reset from four years (2017–2021) to eight years (2013–2021). Only cancer cases in individuals who received no cancer-specific diagnosis or treatment during the washout period were considered index cases [25–27] and thus included in our analysis (see Methodological Appendix Part 2 for details). We further excluded patients aged < 18 years or > 100 years at diagnosis. Figure 1 summarizes how the sample was derived. We followed up all patients for lung cancer-specific hospitalizations until one year after the incidence hospitalization (see Supplementary Table S1 in details).

As this study used pre-existing secondary data for analysis, informed consent was waived. This study was approved by the Ethics Committee for Public Health of Shandong University (LL20241105). We followed the Strengthening the Reporting of Observational Studies in Epidemiology (STROBE) reporting guidelines to ensure the reporting of this observational study [28].

### Health insurance status and covariates

Health insurance status was categorized as URRBMI and UEBMI, based on the insurance type recorded in HRFPs. For the fewer than 10% of individuals who showed changes in scheme enrolment, we used the one that covered the bigger share of their hospitalization counts.

Building on previous literature [29], we included patient socio-demographics, clinical and healthcare provider characteristics as covariates. Socio-demographic characteristics included sex, age, marital status, ethnicity and occupation at diagnosis. Clinical characteristics included the histologic type of lung cancer, whether the cancer has metastasized, and non-cancer comorbidities. Patient comorbidities were assessed using the Charlson Comorbidity Index (CCI), as described by Deyo and colleagues (see Supplementary Table S2 for the score for each diagnostic code) [30]. We categorized patients into three groups based on non-cancer CCI score: 0, 1, and 2 or more. We specifically focused on non-cancer comorbidities to examine their impacts on the lung cancer care [31]. Meanwhile, we obtained information about the level of hospital (tertiary, secondary, and unclassified or other) from the official database of China’s National Health Commission (https://zgcx.nhc.gov.cn). The sites of hospitals were categorized into four regions based on proximity in geography and economic development.

### Outcomes

Our main outcome variables include inpatient cancer care and expenditures within one year after the index hospitalization, because the intensity of medical treatment and expenditures for cancer patients are significantly higher in the first year compared to the years afterwards [32, 33], and that in the first year after diagnosis has a substantial impact on the prognosis of lung cancer [34, 35].

We examined a range of inpatient cancer care variables, including surgery, radiation therapy or chemotherapy, targeted therapy, and immunotherapy identified via a series of ICD-10 and ICD-9-CM3 codes [36] (see Supplementary Table S3), as well as discharge against medical advice (DAMA). DAMA refers to the practice where a patient chooses to leave the hospital against medical assessment based on the patient’s conditions [37]. It is associated with higher risks of morbidity and mortality [38]; therefore, we included it as a key indicator of disparities in cancer care. Besides, we assessed expenditures, including total, out-of-pocket, surgical, drug, and diagnostic expenditures. The expenditures have been adjusted for Consumer Price Index (CPI) using 2021 as a reference level. 

### Statistical analysis

Categorical variables are reported as frequencies (%) while continuous variables are summarized as means with standard deviation (SD). Pearson’s Chi-square test was used for assessing differences of categorical variables.

To control for differences between beneficiaries of the two main categories of SHI, we established propensity score-matched samples (see Methodological Appendix Part 4). One-to-one propensity score matching (PSM) was performed using calipers of width equal to 0.2 of the standard deviation of the logit of the propensity score [39]. Logistic regression was used to calculate a propensity score, which evaluates confounding by indication and/or baseline covariates between two insurance groups. The matching variables used in the PSM models were year of diagnosis, age group, gender, race, marital status, and occupation.

The primary analytical approach was multivariable regression analysis of the propensity score-matched sample (see Methodological Appendix Part 3 for details). We utilized multivariable logistic regressions to measure associations between health insurance status and the receipt of treatment, and conducted subgroup analyses based on whether the cancer had metastasized. We reported average marginal effects, which are interpreted as average differences in the probability of receiving any type of treatment had a beneficiary of URRBMI been covered by UEBMI [40]. Then, we applied a generalized linear model (GLM) with a gamma distribution and log link function to estimate the difference in expenditure attributed to health insurance status. All models were adjusted for covariates mentioned above. We also incorporated the timing of cancer diagnosis by considering fixed effects of the year of diagnosis. Additionally, expenditures were log transformed after adding 1 to all values to allow for zeros.

P values were 2-sided with *P* < 0.05 considered indicative of statistical significance. All statistical analyses were performed using R version 4.3.1.

## Results

### Patient characteristics and matching

319,677 patients were diagnosed with lung cancer between Jan 1, 2017, and Dec 31, 2021, of which 60.3% were insured by URRBMI and 30.5% were insured by UEBMI. The mean age of those covered by URRBMI was 65.5 (SD, 10.1) years, with males accounting for 58.7%. The mean age of UEBMI beneficiaries was 63.5 (SD, 11.6) years, with males accounting for 62.9%. Table 1 shows descriptive statistics for the unmatched and matched samples.

**Table 1:** Descriptive statistics for the study sample, 2017–2021

		Full sample^2^			Propensity score–matched sample		
		No. (%)^3^			No. (%)^3^		
	Overall^1^	URRBMI	UEBMI	SMD^4^	URRBMI	UEBMI	SMD^4^
Characteristic	(*N* = 319677)	(*N* = 192684)	(*N* = 97639)		(*N* = 58389)	(*N* = 58389)	
**Matching Variables**							
Year of diagnosis							
2017	48,010 (15.0)	28,577 (14.8)	14,247 (14.6)	-0.0024	9303 (15.9)	10,850 (18.6)	0.0265
2018	55,579 (17.4)	32,906 (17.1)	16,716 (17.1)	0.0004	10,667 (18.3)	9161 (15.7)	-0.0258
2019	64,703 (20.2)	38,147 (19.8)	20,059 (20.5)	0.0075	12,033 (20.6)	13,378 (22.9)	0.0230
2020	70,761 (22.1)	42,973 (22.3)	21,884 (22.4)	0.0011	13,258 (22.7)	12,738 (21.8)	-0.0089
2021	80,624 (25.2)	50,081 (26.0)	24,733 (25.3)	-0.0066	13,128 (22.5)	12,262 (21.0)	-0.0148
Age at diagnosis							
Mean (SD), years	64.8 (10.7)	65.5 (10.1)	63.5 (11.6)		63.7 (10.8)	63.3 (11.2)	
< 45	10,932 (3.4)	4362 (2.3)	5418 (5.5)	0.0329	2329 (4.0)	1931 (3.3)	-0.0068
45–59	83,109 (26.0)	46,563 (24.2)	28,787 (29.5)	0.0532	17,703 (30.3)	20,987 (35.9)	0.0562
60–75	175,602 (54.9)	111,920 (58.1)	48,424 (49.6)	-0.0849	30,079 (51.5)	26,488 (45.4)	-0.0615
> 75	50,034 (15.7)	29,839 (15.5)	15,010 (15.4)	-0.0011	8278 (14.2)	8983 (15.4)	0.0121
Gender							
Male	191,603 (59.9)	113,187 (58.7)	61,383 (62.9)		35,738 (61.2)	36,078 (61.8)	
Female	128,074 (40.1)	79,497 (41.3)	36,256 (37.1)	-0.0413	22,651 (38.8)	22,311 (38.2)	-0.0058
Ethnicity							
Han	316,257 (98.9)	190,634 (98.9)	96,725 (99.1)		57,872 (99.1)	57,741 (98.9)	
Other	3420 (1.1)	2050 (1.1)	914 (0.9)	-0.0013	517 (0.9)	648 (1.1)	0.0022
Marital status							
Single	7227 (2.3)	4985 (2.6)	1469 (1.5)	-0.0108	631 (1.1)	1215 (2.1)	0.0100
Married	305,317 (95.5)	183,039 (95.0)	94,328 (96.6)	0.0161	56,906 (97.5)	56,112 (96.1)	-0.0136
Divorced	7133 (2.2)	4660 (2.4)	1842 (1.9)	-0.0053	852 (1.5)	1062 (1.8)	0.0036
Occupation							
Employees/workers	29,829 (9.3)	7292 (3.8)	20,224 (20.7)	0.1693	7292 (12.5)	5988 (10.3)	-0.0223
Non-practitioners^5^	158,322 (49.5)	130,156 (67.5)	16,045 (16.4)	-0.5112	16,045 (27.5)	16,045 (27.5)	0.0000
Special Employees^6^	39,191 (12.3)	5130 (2.7)	30,833 (31.6)	0.2892	5130 (8.8)	6990 (12.0)	0.0319
Unspecified	92,335 (28.9)	50,106 (26.0)	30,537 (31.3)	0.0527	29,922 (51.2)	29,366 (50.3)	-0.0095
**Non-Matching Variables**							
Types of lung cancer				< 0.001			< 0.001
SCLC	29,934 (9.4)	20,651 (10.7)	7439 (7.6)		6065 (10.4)	4589 (7.9)	
NSCLC	203,011 (63.5)	115,477 (59.9)	68,673 (70.3)		37,296 (63.9)	40,115 (68.7)	
Unspecified	86,732 (27.1)	56,556 (29.4)	21,527 (22.0)		15,028 (25.7)	13,685 (23.4)	
Tumor metastasis				< 0.001			< 0.001
No	250,591 (78.4)	146,405 (76.0)	80,374 (82.3)		45,469 (77.9)	47,741 (81.8)	
Yes	69,086 (21.6)	46,279 (24.0)	17,265 (17.7)		12,920 (22.1)	10,648 (18.2)	
CCI				< 0.05			< 0.001
CCI = 0	198,764 (62.2)	119,260 (61.9)	60,608 (62.1)		37,088 (63.5)	36,535 (62.6)	
CCI = 1	81,587 (25.5)	49,762 (25.8)	24,782 (25.4)		14,592 (25.0)	14,687 (25.2)	
CCI > = 2	39,326 (12.3)	23,662 (12.3)	12,249 (12.5)		6709 (11.5)	7167 (12.3)	
Hospital level				< 0.001			< 0.001
Secondary hospitals	82,728 (25.9)	59,222 (30.7)	15,840 (16.2)		13,244 (22.7)	9633 (16.5)	
Tertiary hospitals	234,941 (73.5)	132,318 (68.7)	81,350 (83.3)		44,819 (76.8)	48,468 (83.0)	
Unclassified or other	2008 (0.6)	1144 (0.6)	449 (0.5)		326 (0.6)	288 (0.5)	
Hospital region^7^				< 0.001			< 0.001
Eastern (Peninsula) Region	74,962 (23.4)	32,515 (16.9)	32,765 (33.6)		18,446 (31.6)	21,999 (37.7)	
Northern Region	41,561 (13.0)	30,494 (15.8)	8761 (9.0)		6122 (10.5)	4768 (8.2)	
Southern Region	80,323 (25.1)	60,804 (31.6)	12,869 (13.2)		12,582 (21.5)	8092 (13.9)	
Central Region	122,831 (38.4)	68,871 (35.7)	43,244 (44.3)		21,239 (36.4)	23,530 (40.3)	

Note. Abbreviations: UEBMI, Urban Employee Basic Medical Insurance; URRBMI, Urban and Rural Residents Basic Medical Insurance; NSCLC, non-small cell lung cancer. SCLC, Small cell lung cancer; CCI, the Charlson Comorbidity Index; SMD, standardized mean difference (absolute value of difference in means divided by the standard deviation)

^1^Other insurance types (e.g., public health insurance, private health insurance, supplementary health insurance, poverty assistance, etc.) and no insured patients (i.e., all paid out of pocket) represented 6.9% and 2.3% of the cohort and are not included in this table

^2^Sample is drawn from the overall data of URRBMI and UEBMI populations and is used for analyzing treatments and expenditures

^3^Values are written as No. (%) unless otherwise stated

^4^SMD is presented for matching variables as the PSM result, while P-value from Pearson’s Chi-square test is shown for non-matching variables to evaluate intergroup difference significance

^5^Non-practitioners: Self-employed /Unemployed/ Freelance/Students /Farmers

^6^Special Employees: Retired (retired) staff/civil servants/Professional and technical staff

^7^Based on the topography, population and culture of Shandong Province, the hospital regions are divided into four regions: the Jiaodong Peninsula region (Eastern (Peninsula) Region), the Luzhong region (Central Region), the Lubei region (Northern Region) and the Lunan region (Southern Region)

Socio-demographic characteristics across the 2 matched groups were well balanced, with all standardized mean differences smaller than 0.1. After matching, 68.7% of UEBMI patients were diagnosed with NSCLC, and 12.3% had CCI ≥ 2, both higher than the 63.9% and 11.5% observed in URRBMI patients. Furthermore, UEBMI patients were more likely to receive treatment at tertiary hospitals (83.0% vs. 76.8%) and at hospitals located in the Central Region (40.3% vs. 36.4%). Notably, 22.1% of URRBMI patients had tumor metastasis, which was higher than the 18.2% occurred among UEBMI patients.

### Cancer treatment disparity after PSM

**Fig. 2:**
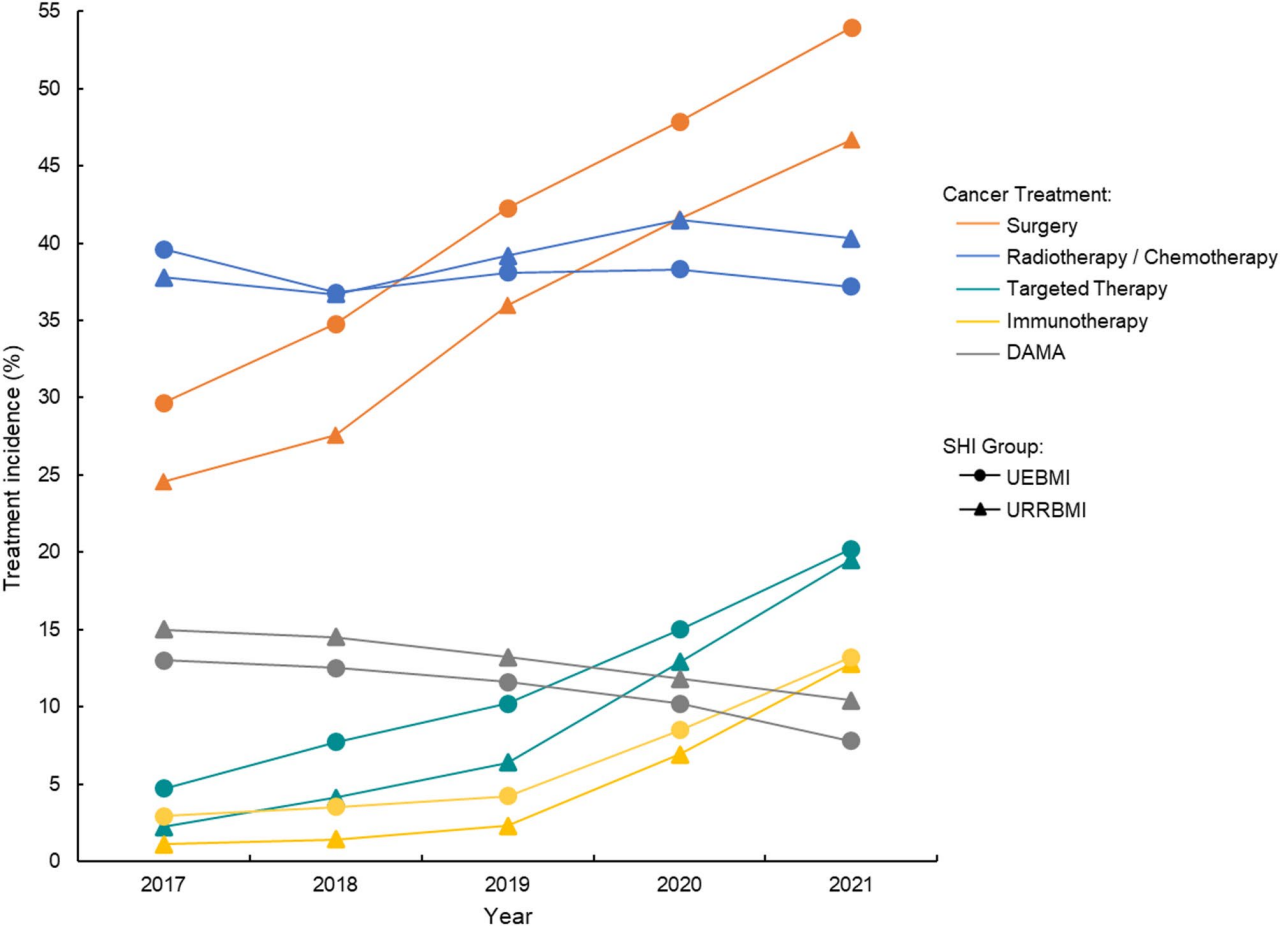
Trends in incidence of inpatient lung cancer treatments among URRBMI and UEBMI beneficiaries in China from 2017 to 2021^1^. *Note* Abbreviations: SHI, social health insurance; UEBMI, Urban Employee Basic Medical Insurance; URRBMI, Urban and Rural Residents Basic Medical Insurance; DAMA, discharge against medical advice. ^1^Samples contain propensity score–matched lung cancer patients in 2017-21

Figure 2 demonstrates trends in inpatient cancer treatment among URRBMI and UEBMI beneficiaries diagnosed with lung cancer. From 2017 to 2021, there were increasing proportions of matched samples receiving surgery (UEBMI: 29.7% in 2017 to 54.0% in 2021; URRBMI: 24.6% in 2017 to 46.7% in 2021), targeted therapy (UEBMI: 4.7% in 2017 to 20.2% in 2021; URRBMI: 2.2% in 2017 to 19.5% in 2021), and immunotherapy (UEBMI: 2.9% in 2017 to 13.2% in 2021; URRBMI: 1.1% in 2017 to 12.8% in 2021), with the gradually decreasing difference between the two schemes. Meanwhile, the proportions of DAMA were decreasing among matched samples in both groups (UEBMI: 13.0% in 2017 to 7.8% in 2021; URRBMI: 15.0% in 2017 to 10.4% in 2021).

Table 2 displays cancer-directed inpatient treatment and the average marginal effects of UEBMI beneficiaries over URRBMI beneficiaries on the likelihood of receiving cancer therapy for the matched sample. UEBMI beneficiaries were more likely, compared with URRBMI beneficiaries, to receive surgery (46.0% vs. 34.7%), targeted therapy (11.5% vs. 9.6%), and immunotherapy (6.4% vs. 5.4%). According to the results of the multivariable logistic regression, UEBMI was associated with increased probabilities of receiving surgery (average marginal difference [AME]: 6.76%; 95% CI, 6.31 to 7.20%), targeted therapy (AME, 2.39%; 95% CI, 2.05 to 2.73%) and immunotherapy (AME, 1.26%; 95% CI, 1.00 to 1.53%). In contrast, UEBMI was associated with lower probabilities to experience chemotherapy or radiotherapy (AME, -0.55%; 95% CI, -1.09 to -0.02%) and DAMA (AME, -1.56%; 95% CI, -1.92 to -1.20%).

**Table 2:** Differences in lung cancer treatment in the first year after diagnosis, by health insurance schemes, 2017-21

Treatment Outcomes	No. (%)		UEBMI vs. URRBMI^1^	
	UEBMI (*N* = 58389)	URRBMI (*N* = 58389)	AME [95% CI] (%)^2^	*P*-value
Surgery			6.76 [6.31, 7.20]	< 0.001
Yes	26,866 (46.0)	20,253 (34.7)		
No	31,523 (54.0)	38,136 (65.3)		
Radiotherapy / Chemotherapy			-0.55 [-1.09, -0.02]	< 0.05
Yes	21,890 (37.5)	22,725 (38.9)		
No	36,499 (62.5)	35,664 (61.1)		
Targeted Therapy			2.39 [2.05, 2.73]	< 0.001
Yes	6731 (11.5)	5623 (9.6)		
No	51,658 (88.5)	52,766 (90.4)		
Immunotherapy			1.26 [1.00, 1.53]	< 0.001
Yes	3759 (6.4)	3132 (5.4)		
No	54,630 (93.6)	55,257 (94.6)		
Discharge Against Medical Advice			-1.56 [-1.92, -1.20]	< 0.001
Yes	6013 (10.3)	7467 (12.8)		
No	52,376 (89.7)	50,922 (87.2)		

Note. Abbreviations: UEBMI, Urban Employee Basic Medical Insurance; URRBMI, Urban and Rural Residents Basic Medical Insurance. AME, Adjusted Marginal Effect

^1^Models adjust for patient socio-demographics, year of diagnosis, clinical characteristics and healthcare provider characteristics. Treatment outcomes were analyzed using a multivariate regression analysis

^2^ Values for this column are rounded to two decimal places

### Subgroups analysis of cancer treatment disparity

Table 3 presents the cancer-directed inpatient treatment and the AMEs of UEBMI over URRBMI on the likelihood of receiving cancer therapy for matched subgroups, categorized by the presence or absence of metastasis. Distribution of the propensity scores for the unmatched and matched subgroups is provided in Supplementary Tables S4-5.

**Table 3:** Comparison of treatment between URRBMI and UEBMI in Non-metastatic and metastatic lung Cancer patients, 2017–2021

**Treatment**	**No. (%)**		**UEBMI vs. URRBMI** ^**2**^	
	**UEBMI** **(** ***N*** **= 47170)**	**URRBMI** **(** ***N*** **= 47170)**	**AME [95% CI] (%)** ^**3**^	***p*** **-value**
**Non-metastatic Group** ^**1**^				
Surgery			8.04 [7.52, 8.57]	< 0.001
Yes	26,916 (57.1)	20,482 (43.4)		
No	20,254 (42.9)	26,688 (56.6)		
Radiotherapy / Chemotherapy			-1.40 [-1.98, -0.82]	< 0.001
Yes	15,811 (33.5)	17,114 (36.3)		
No	31,359 (66.5)	30,056 (63.7)		
Targeted Therapy			1.16 [0.82, 1.50]	< 0.001
Yes	3983 (8.4)	3395 (7.2)		
No	43,187 (91.6)	43,775 (92.8)		
Immunotherapy			0.86 [0.59, 1.14]	< 0.001
Yes	2572 (5.5)	2190 (4.6)		
No	44,598 (94.5)	44,980 (95.4)		
Discharge Against Medical Advice			-1.79 [-2.16, -1.41]	< 0.001
Yes	3971 (8.4)	5349 (11.3)		
No	43,199 (91.6)	41,821 (88.7)		
	**No. (%)**		**UEBMI vs. URRBMI** ^**2**^	
**Treatment**	**UEBMI** **(** ***N*** **= 11298)**	**URRBMI** **(** ***N*** **= 11298)**	**AME [95% CI] (%)** ^**3**^	**p-value**
**Metastatic Group** ^**1**^				
Surgery			1.13 [0.52, 1.74]	< 0.001
Yes	732 (6.5)	571 (5.1)		
No	10,566 (93.5)	10,727 (94.9)		
Radiotherapy / Chemotherapy			3.49 [2.27, 4.71]	< 0.001
Yes	5873 (52.0)	5352 (47.4)		
No	5425 (48.0)	5946 (52.6)		
Targeted Therapy			7.87 [6.89, 8.86]	< 0.001
Yes	2893 (25.6)	1967 (17.4)		
No	8405 (74.4)	9331 (82.6)		
Immunotherapy			3.41 [2.69, 4.14]	< 0.001
Yes	1222 (10.8)	849 (7.5)		
No	10,076 (89.2)	10,449 (92.5)		
Discharge Against Medical Advice			-1.15 [-2.14, -0.16]	< 0.05
Yes	1900 (16.8)	2099 (18.6)		
No	9398 (83.2)	9199 (81.4)		

Note. Abbreviations: UEBMI, Urban Employee Basic Medical Insurance; URRBMI, Urban and Rural Residents Basic Medical Insurance. AME, Adjusted Marginal Effect

^1^This table is based on two subgroup samples including the presence or absence of cancer metastases. Descriptive statistics for the samples are provided in Appendix Table

^2^Models adjust for patient socio-demographics, year of diagnosis, clinical characteristics and healthcare provider characteristics. Treatment outcomes were analyzed using a multivariate regression analysis

^3^ Values for this column are rounded to two decimal places

In patients without metastasis, UEBMI was associated with a higher rate of surgery (AME, 8.04%; 95% CI, 7.52 to 8.57%) but a lower rate of chemotherapy or radiotherapy alone (AME, -1.40%; 95% CI, -1.98% to -0.82%). Among patients with metastasis, the AME of UEBMI was 1.13% (95% CI, 0.52 to 1.74%) for surgery, and 3.49% (95% CI, 2.27 to 4.71%) for chemotherapy or radiotherapy. Additionally, in both subgroups, UEBMI beneficiaries were consistently associated with higher likelihoods of receiving both targeted therapy and immunotherapy (particularly among the group with metastatic cancer), and a lower likelihood of DAMA (non-metastatic group: AME, -1.79%; 95% CI, -2.16 to -1.41%; metastatic group: AME, -1.15%; 95% CI, -2.14 to -0.16%).

### Disparity in expenditures after PSM

Table 4 presents differences in inpatient expenditures for the matched sample of lung cancer patients under URRBMI and UEBMI(see Supplementary Table S6 for further descriptive statistics about the unmatched and matched samples). Patients under UEBMI had 13.34% higher total expenditures compared with those under URRBMI (95% CI, 13.08 to 15.47%). Specifically, surgical expenditures were 18.57% higher for UEBMI patients (95% CI, 17.68 to 23.19%), drug expenditures were 8.21% higher (95% CI, 6.87 to 10.26%), and diagnostic expenditures were 8.21% higher (95% CI, 6.90 to 10.25%). However, out-of-pocket expenditures for UEBMI patients was 19.09% lower than for URRBMI patients (95% CI, -18.39 to -16.36%).

**Table 4:** Differences in medical expenditure in the first year after diagnosis, by health insurance schemes, 2017 − 21^1^

Expenditures, RMB	Adjusted mean (95%CI)		GLM results (UEBMI vs. URRBMI)^2^	
	URRBMI^1^ (*N* = 40820)	UEBMI^1^ (*N* = 40820)	Exp(coefficient)-1 [95% CI] (%)^3^	*P*-value
Total expenditures	60,383.08 (59,868.90–60,897.26)	69,812.93 (69,268.91–70,356.94)	13.34 [13.08, 15.47]	< 0.001
Out-of-pocket expenditures	33,258.46 (32,940.02–33,576.90)	27,193.36 (26,921.38–27,465.35)	-19.09 [-18.39, -16.36]	< 0.001
Surgical expenditures	7,929.47 (7,793.81–8,065.13)	9,606.28 (9,466.10–9,746.46)	18.57 [17.68, 23.19]	< 0.001
Drug expenditures	18,157.26 (17,906.03–18,408.48)	19,963.04 (19,679.57–20,246.51)	8.21 [6.87, 10.26]	< 0.001
Diagnosis-related expenditures	14,828.55 (14,700.75–14,956.34)	15,420.04 (15,298.49–15,541.60)	8.21 [6.90, 10.25]	< 0.001

Note. Abbreviations: UEBMI, Urban Employee Basic Medical Insurance; URRBMI, Urban and Rural Residents Basic Medical Insurance. GLM, generalized linear model

^1^This table is based on a PSM sample that excludes cases with zero or missing medical expenditure. Descriptive statistics for the sample are provided in Appendix Table

^2^Models adjust for patient socio-demographics, year of diagnosis, clinical characteristics and healthcare provider characteristics. Expenditures outcomes were analyzed using a GLM with a gamma distribution and log link, with outcomes in 2021 inflation-adjusted terms

^3^ Exp (coefficient) -1 (%) reflects the relative change proportion of the medical expenditures in the UEBMI group compared to the URRBMI group. Values for this column are rounded to two decimal places

## Discussion

Taking advantage of population-wide discharge data from one of the most populous provinces in China, we analyzed the inequalities between URRBMI and UEBMI beneficiaries in inpatient lung cancer treatment and related expenditures. Using the PSM method to adjust for different patient characteristics across the two schemes, we observed an increase in utilization of treatment services for lung cancer patients in Shandong, China. However, significant inequalities remained in both cancer treatment and financial protection between URRBMI and UEBMI beneficiaries in China, with a notable disparity in surgical treatment, particularly among those without metastatic cancer.

Our findings about the treatment inequalities between cancer patients under different SHI schemes in China, particularly in terms of surgery, are consistent with findings from multiple existing studies showing more generous insurance to be associated with higher rates of receiving cancer treatment (especially curative surgery) [41–44]. Crucially, we found the inequalities in surgery to be more pronounced among patients with non-metastatic lung cancer. Similar findings have been reported in the United States, where uninsured and Medicaid patients were less likely to receive surgery among patients with early-stage lung cancer compared with Medicare patients [45, 46]. Comparable studies also identified discrepancies in delays of care and receipt of resection related to insurance within pancreatic, colorectal and hepatocellular cancers [47–49]. As early surgical intervention is associated with significantly longer survival for lung cancer [50], the treatment inequalities in early-stage cancer likely translate into inequalities in survival. Meanwhile, the observed rapid increase in cancer treatment rates, with surgical rates nearly doubling during the study period, may reflect improved access to surgical care and the effectiveness of cancer screening programs in China. However, this trend could also suggest the possibility of overdiagnosis or overuse of surgery, a hypothesis that has not been investigated in this study and warrants further research.

The centralized procurement of innovative anticancer drugs by China’s National Healthcare Security Administration since 2018 led to an increasing number of receiving SHI reimbursement, which likely contributed to the rising proportion of people receiving targeted therapy or immunotherapy observed in our study. Particularly among patients with metastatic lung cancer, the main care advantage associated with UEBMI in comparison to URRBMI shifted to the utilization of radiotherapy, chemotherapy, targeted therapy and immunotherapy.

Previous studies also found the type of health insurance might affect patients’ treatment choices and the continuity of care they receive [51]. Consistent with these findings, our study found that URRBMI (with UEBMI as reference) was associated with a higher rate of DAMA, which might result in rapid deterioration of the disease and shortened survival time [52]. Similar results have been observed in stage IV non-small-cell lung cancer patients, where Medicare and uninsured patients were more likely to refuse treatment compared to those with private insurance [29]. Additionally, we observed that URRBMI patients receive care at secondary hospitals, which may have limited medical resources compared to tertiary hospitals frequented by UEBMI patients. This reliance, influenced by higher cost-sharing for tertiary care in urban areas [53], may exacerbate disparities in clinical outcomes due to systemic inequities in care quality.

In our study, the persisted disparities in lung cancer care across SHI schemes after controlling for potential confounders, reveal potentially substantial unwarranted variations in cancer that could not be explained by illness severity or patient preference [14, 54].Besides factors on the demand-side, potential explanation from the provider perspectives is that physicians adjust their clinical management in response to patients’ insurance schemes [55] that provide differed financial incentives and constraints. Specifically, the more generous reimbursement rates of UEBMI may encourage physicians to take more aggressive cancer care. Empirical evidence supports this, showing physicians respond to financial incentives by changing prescribing behavior [56], or elective procedure use [57].

In terms of expenditures, patients under UEBMI experienced better financial protection than those under URRBMI, which is consistent with previous research [58, 59]. This economic advantage appears to translate into disparities in cancer healthcare, where UEBMI patients demonstrated greater access to cancer care than their URRBMI patients [5]. The heightened financial burden faced by URRBMI beneficiaries carries particular implications for vulnerable populations. Moreover, a previous study [60] demonstrated that cancer patients over 60 years old faced a heavier financial burden, with high hospitalization costs potentially becoming a barrier for the elderly.

### Implications for policy and practice

Several implications can be drawn for this study. First, the double inequalities in treatment and financial protection for lung cancer patients imply that inadequacy in cancer care for the URRBMI beneficiaries. To improve access, especially for non-metastatic or early-stage lung cancer patients, it is crucial to consider narrowing the reimbursement gaps for surgery between URRBMI and UEBMI, enabling URRBMI beneficiaries to afford necessary surgery at the right stage and avoid catastrophic expenditure. Indeed, policies on benefit packages and reimbursement rates may be further coordinated, so that incremental harmonization of SHI schemes prioritize raising reimbursement rates for good value cancer care. Additionally, clear clinical guidelines and oversight mechanisms are necessary to prevent resource misuse and mitigate moral hazard. Second, the disparity in radiotherapy/chemotherapy, targeted therapy and immunotherapy in (particularly metastatic) lung cancer patients across SHI schemes in China should also raise discussions about standardization of cancer care and “value for money”. It is possible that some expenditures of UEBMI in the late-stage cancer care could be made to much better use in URRBMI for patients with an earlier stage cancer. Third, given that a much larger proportion of URRBMI patients receive care at the secondary hospitals than UEBMI patients, it is critical to enhance the quality and continuity of medical services at secondary hospitals to minimize disparities in care quality relative to tertiary hospitals.

### Limitations

Several limitations of our study warrant caution in interpretation. First, there is an absence of comprehensive staging information for lung cancer patients in our dataset. While this precluded a more nuanced analysis of how detailed stages of lung cancer affect outcomes across SHI schemes, our subgroup analysis stratified by whether the cancer was metastatic should have addressed a substantial part of the patient’s cancer stage upon diagnosis. Second, limited socio-economic data prevented us from distinguishing the effects of SHI scheme from confounders like health literacy, attitudes towards surgery, and social or family support. Lower health literacy and risk-averse attitudes toward surgery may reduce treatment adherence and uptake of aggressive treatments [61, 62], while stronger social support likely enhances treatment-seeking [61]. Third, while our study did not delve into the quality or health outcome of treatment received, or whether the unwarranted disparity represents under- or over-treatment, these aspects present important avenues for future research. Fourth, given the mobility of patients, the HRFPs would not capture hospitalizations outside Shandong Province, which means an underestimation of treatments and expenditures. However, due to the province’s large population and well-developed healthcare resources, cancer patients often rely heavily on local care. Hence, this underestimation is likely to be small. Finally, the data were obtained from medical institutions in Shandong Province means our results are not directly generalizable to other provinces or countries. However, the significant disparities we found may have some reference value for understanding the situation in China, as the tiered SHI exists nation-wide.

## Conclusion

Utilization of inpatient cancer treatment services improved in recent years for lung cancer patients under both UEBMI and URRBMI in China. Compared to URRBM, UEBMI coverage was associated with a substantially higher likelihood of receiving surgery, radiotherapy, and chemotherapy, especially surgery among patients with non-metastatic cancer. They also experienced lower out-of-pocket expenditures, indicating better financial protection. These findings highlight the need to better harmonize benefits within China’s tiered SHI system to reduce disparities in cancer care.

## Electronic supplementary material

Below is the link to the electronic supplementary material.

Supplementary Material 1

## Data Availability

No datasets were generated or analysed during the current study.

## References

[1] World Health Organization. The World Health Report: health systems financing: the path to universal coverage: executive summary [Internet]. Geneva: WHO. 2012 Jun 16 [cited 2024 Nov 1]. Available for download from: https://www.who.int/publications/i/item/9789241564021 Google Scholar.

[2] Angell B, Dodd R, Palagyi A, et al. Primary health care financing interventions: a systematic review and stakeholder-driven research agenda for the Asia-Pacific region. BMJ Glob Health. 2019;4(Suppl 8):e001481. 10.1136/bmjgh-2019-001481. 31478024 10.1136/bmjgh-2019-001481 PMC6703289

[3] Appleby J, Raleigh V, Frosini F, Bevan G, Gao H, Lyscom T. Variations in health care: the good, the bad and the inexplicable. In:; 2011. Accessed September 25, 2024. https://www.kingsfund.org.uk/sites/default/files/Variationsin-health-care-good-bad-inexplicable-report-The-Kings-Fund-April-2011. pdf.

[4] Johnson A, Stukel T, editors. Medical practice variations. In: Springer US; 2016. 10.1007/978-1-4899-7573-7.

[5] Witthayapipopsakul W, Viriyathorn S, Rittimanomai S, et al. Health insurance schemes and their influences on healthcare variation in Asian countries: A realist review and theory’s testing in Thailand. Int J Health Policy Manag Published Online Febr. 2024;17:1. 10.34172/ijhpm.2024.7930. 10.34172/ijhpm.2024.7930 PMC11608294 39099526

[6] Fang H, Eggleston K, Hanson K, Wu M. Enhancing financial protection under China’s social health insurance to achieve universal health coverage. BMJ. 2019;365. 10.1136/bmj.l2378. 10.1136/bmj.l2378 PMC6598720 31227485

[7] Zhao Y, Zhang L, Fu Y, Wang M, Zhang L. Socioeconomic disparities in Cancer treatment, service utilization and catastrophic health expenditure in China: A Cross-Sectional analysis. Int J Environ Res Public Health. 2020;17(4):1327. 10.3390/ijerph17041327. 32092913 10.3390/ijerph17041327 PMC7068279

[8] Li D, Lei HK, Shu XL et al. Association of public health insurance with cancer-specific mortality risk among patients with nasopharyngeal carcinoma: a prospective cohort study in China. *Frontiers in Public Health*. 2023;11. Accessed February 29, 2024. https://www.frontiersin.org/journals/public-health/articles/10.3389/fpubh.2023.1020828 10.3389/fpubh.2023.1020828 PMC10272587 37333541

[9] Li X, Zhou Q, Wang X, et al. The effect of low insurance reimbursement on quality of care for non-small cell lung cancer in China: a comprehensive study covering diagnosis, treatment, and outcomes. BMC Cancer. 2018;18(1):683. 10.1186/s12885-018-4608-y. 29940893 10.1186/s12885-018-4608-y PMC6019825

[10] Su S, Bao H, Wang X, et al. The quality of invasive breast cancer care for low reimbursement rate patients: A retrospective study. PLoS ONE. 2017;12(9). 10.1371/journal.pone.0184866. 10.1371/journal.pone.0184866 PMC5599036 28910357

[11] Wang Y, Lei H, Li X, et al. Lung Cancer-Specific mortality risk and public health insurance: A prospective cohort study in Chongqing, Southwest China. Front Public Health. 2022;10:842844. 10.3389/fpubh.2022.842844. 35570974 10.3389/fpubh.2022.842844 PMC9099244

[12] National Healthcare Security Administration, National Healthcare Security Development Statistical Bulletin. (2024, July 25). 2023. Retrieved January 8, 2025, from https://www.nhsa.gov.cn/art/2024/7/25/art_7_13340.html

[13] China Statistical Yearbook. 2024. Accessed December 22, 2024. https://www.stats.gov.cn/sj/ndsj/2024/indexeh.htm

[14] Wennberg JE. Time to tackle unwarranted variations in practice. BMJ. 2011;342:d1513. 10.1136/bmj.d1513. 21415111 10.1136/bmj.d1513

[15] Dwyer LL, Vadagam P, Vanderpoel J, Cohen C, Lewing B, Tkacz J. Disparities in lung cancer: A targeted literature review examining lung Cancer screening, diagnosis, treatment, and survival outcomes in the united States. J Racial Ethn Health Disparities. 2024;11(3):1489–500. 10.1007/s40615-023-01625-2 37204663 10.1007/s40615-023-01625-2 PMC11101514

[16] Marlow N, Pavluck A, Bian J, Halpern M. The relationship between insurance coverage and Cancer care: A literature synthesis. RTI Press, May 2009. p. 16-20. 10.3768/rtipress.2009.rr.0005.0905 31216155

[17] Deng P, Fu Y, Chen M, Si L. Factors associated with health care utilization and catastrophic health expenditure among cancer patients in China: evidence from the China health and retirement longitudinal study. Front Public Health. 2022;10:943271. 10.3389/fpubh.2022.943271. 36438282 10.3389/fpubh.2022.943271 PMC9684646

[18] Sun C, yao, Shi J fang, Fu W et al. qi,. Catastrophic health expenditure and its determinants in households with lung cancer patients in China: a retrospective cohort study. *BMC Cancer*. 2021;21(1):1323. 10.1186/s12885-021-09030-w 10.1186/s12885-021-09030-w PMC8665572 34893037

[19] Fu W, Shi J, Liu C, Chen W, Liu G, He J. Health insurance and inequalities in catastrophic health spending in cancer patients. A cross-sectional study in China. Gac Sanit. 2024;38:102397. 10.1016/j.gaceta.2024.102397. 38772059 10.1016/j.gaceta.2024.102397

[20] Han B, Zheng R, Zeng H, et al. Cancer incidence and mortality in China, 2022. J Natl Cancer Cent. 2024;4(1):47–53. 10.1016/j.jncc.2024.01.006. 39036382 10.1016/j.jncc.2024.01.006 PMC11256708

[21] Bray F, Laversanne M, Sung H et al. Global cancer statistics 2022: GLOBOCAN estimates of incidence and mortality worldwide for 36 cancers in 185 countries. *CA: A Cancer Journal for Clinicians*. n/a(n/a). 10.3322/caac.21834 10.3322/caac.21834 38572751

[22] Chen S, Cao Z, Prettner K, et al. Estimates and projections of the global economic cost of 29 cancers in 204 countries and territories from 2020 to 2050. JAMA Oncol. 2023;9(4):465–72. 10.1001/jamaoncol.2022.7826. 36821107 10.1001/jamaoncol.2022.7826 PMC9951101

[23] Shandong Provincial Health Commission. Report on the Incidence and Mortality of Key Chronic Diseases in Shandong Province. (2018) [Internet]. 2020 Dec 16 [Cited 2025 Apr 20]. Available from: http://Wsjkw.Shandong.Gov.Cn/Zwgk/Fdzdgknr/Tjgb/202012/P020201216405805597738.Pdf.

[24] National Bureau of Statistics of China. China Statistical Yearbook. 2024. National Bureau of Statistics of China. https://Www.Stats.Gov.Cn/Sj/Ndsj/2024/Indexeh.Htm. Published 2024. Accessed January 16, 2025.

[25] Czwikla J, Jobski K, Schink T. The impact of the lookback period and definition of confirmatory events on the identification of incident cancer cases in administrative data. BMC Med Res Methodol. 2017;17(1):122. 10.1186/s12874-017-0407-4. 28806932 10.1186/s12874-017-0407-4 PMC5556662

[26] Wenyi Y, Jingxin W, Limei AI, Xia W. a. N. Optimal strategies for determining the duration of washout period in the context of identifying chronic disease onset cases based on administrative data: a systematic review. Chin Gen Pract. 2024;27(04):460. 10.12114/j.issn.1007-9572.2023.0005.

[27] Ni X, Li Z, Li X, et al. Socioeconomic inequalities in cancer incidence and access to health services among children and adolescents in China: a cross-sectional study. Lancet. 2022;400(10357):1020–32. 10.1016/S0140-6736(22)01541-0. 36154677 10.1016/S0140-6736(22)01541-0

[28] Equator Network. STROBE Checklist Cohort. Accessed June 27. 2024. https://www.equator-network.org/wp-content/uploads/2015/10/STROBE_checklist_v4_cohort.pdf

[29] Duma N, Idossa DW, Durani U, et al. Influence of sociodemographic factors on treatment decisions in Non-Small-Cell lung Cancer. Clin Lung Cancer. 2020;21(3):e115–29. 10.1016/j.cllc.2019.08.005. 31570228 10.1016/j.cllc.2019.08.005

[30] Deyo RA, Cherkin DC, Ciol MA. Adapting a clinical comorbidity index for use with ICD-9-CM administrative databases. J Clin Epidemiol. 1992;45(6):613–9. 10.1016/0895-4356(92)90133-8. 1607900 10.1016/0895-4356(92)90133-8

[31] Hb M, Sd S. Adapting the elixhauser comorbidity index for cancer patients. Cancer. 2018;124(9). 10.1002/cncr.31269. 10.1002/cncr.31269 PMC5910176 29390174

[32] Price RA, Stranges E, Elixhauser A. Healthcare Cost and Utilization Project. Statistical Brief No. 125: Cancer hospitalizations for adults, 2009. Rockville, MD, Agency for Healthcare Research and Quality, 2012. https://www.hcup-us.ahrq.gov/reports/statbriefs/sb125.pdf 22574333

[33] Kishimoto K, Kunisawa, Fushimi K, Imanaka Y. Individual and nationwide costs for Cancer care during the first year after diagnosis among children, adolescents, and young adults in Japan. JCO Oncol Pract. 2022;18(3):e351–9. 10.1200/OP.21.00364. 34570620 10.1200/OP.21.00364

[34] Warren JL, Yabroff KR, Meekins A, Topor M, Lamont EB, Brown ML. Evaluation of trends in the cost of initial cancer treatment. J Natl Cancer Inst. 2008;100(12):888–97. 10.1093/jnci/djn175. 18544740 10.1093/jnci/djn175 PMC3298963

[35] Yabroff KR, Lamont EB, Mariotto A, et al. Cost of care for elderly cancer patients in the united States. J Natl Cancer Inst. 2008;100(9):630–41. 10.1093/jnci/djn103. 18445825 10.1093/jnci/djn103

[36] Qian Y, Jiayi G, Pan Z, Suting Z, Saibin W, Minhui X. Guo Xiaodong. Coding of main diagnosis and treatment procedures for lung cancer. Chin J Hosp Stastics. 2020;27(4):358–61.

[37] Chinese Hospital Association. (2022). China Hospital Quality and Safety Management: Part 1–4—General Principles—Standard General Terms. Retrieved January 8, 2025, from https://Cmsfiles.Zhongkefu.ComCn/Zgyiyuanc/Upload/Zgyiyuan/File/20230821/1692609079885483.Pdf

[38] Holmes EG, Cooley BS, Fleisch SB, Rosenstein DL. Against medical advice discharge: A narrative review and recommendations for a systematic approach. Am J Med. 2021;134(6):721–6. 10.1016/j.amjmed.2020.12.027. 33610522 10.1016/j.amjmed.2020.12.027

[39] Austin PC. Optimal caliper widths for propensity-score matching when estimating differences in means and differences in proportions in observational studies. Pharm Stat. 2011;10(2):150–61. 10.1002/pst.433. 20925139 10.1002/pst.433 PMC3120982

[40] Norton EC, Dowd BE, Maciejewski ML. Marginal effects-Quantifying the effect of changes in risk factors in logistic regression models. JAMA. 2019;321(13):1304–5. 10.1001/jama.2019.1954. 30848814 10.1001/jama.2019.1954

[41] Ubbaonu CD, Chang J, Ziogas A, Mehta RS, Kansal KJ, Zell JA. Disparities in receipt of National comprehensive Cancer network Guideline-Adherent care and outcomes among women with Triple-Negative breast Cancer by race/ethnicity, socioeconomic status, and insurance type. Cancers. 2023;15(23). 10.3390/cancers15235586. 10.3390/cancers15235586 PMC10705726 38067290

[42] Slatore CG, Au DH, Gould MK. An official American thoracic society systematic review: insurance status and disparities in lung Cancer practices and outcomes. Am J Respir Crit Care Med. 2010;182(9):1195–205. 10.1164/rccm.2009-038ST. 21041563 10.1164/rccm.2009-038ST

[43] Karanth S, Fowler ME, Mao X, et al. Race, socioeconomic status, and Health-Care access disparities in ovarian Cancer treatment and mortality: systematic review and Meta-Analysis. JNCI Cancer Spectr. 2019;3(4):pkz084. 10.1093/jncics/pkz084. 31840133 10.1093/jncics/pkz084 PMC6899434

[44] Fonseca AL, Khan H, Mehari KR, Cherla D, Heslin MJ, Johnston FM. Disparities in access to oncologic care in pancreatic cancer: A systematic review. Ann Surg Oncol. 2022;29(5):3232–50. 10.1245/s10434-021-11258-6. 35067789 10.1245/s10434-021-11258-6

[45] Wakeam E, Varghese TK, Leighl NB, Giuliani M, Finlayson SRG, Darling GE. Trends, practice patterns and underuse of surgery in the treatment of early stage small cell lung cancer. Lung Cancer. 2017;109:117–23. 10.1016/j.lungcan.2017.05.004. 28577940 10.1016/j.lungcan.2017.05.004

[46] Stokes SM, Wakeam E, Swords DS, Stringham JR, Varghese TK. Impact of insurance status on receipt of definitive surgical therapy and posttreatment outcomes in early stage lung cancer. Surgery. 2018;164(6):1287–93. 10.1016/j.surg.2018.07.020. 30170821 10.1016/j.surg.2018.07.020

[47] Shapiro M, Chen Q, Huang Q, et al. Associations of socioeconomic variables with resection, stage, and survival in patients with early-stage pancreatic cancer. JAMA Surg. 2016;151(4):338–45. 10.1001/jamasurg.2015.4239. 26581025 10.1001/jamasurg.2015.4239

[48] Mitsakos AT, Irish W, Parikh AA, Snyder RA. The association of health insurance and race with treatment and survival in patients with metastatic colorectal cancer. PLoS ONE. 2022;17(2):e0263818. 10.1371/journal.pone.0263818. 35176030 10.1371/journal.pone.0263818 PMC8853572

[49] Qiu Z, Qi W, Wu Y, Li L, Li C. Insurance status impacts survival of hepatocellular carcinoma patients after liver resection. Cancer Med. 2023;12(16):17037–46. 10.1002/cam4.6339. 37455560 10.1002/cam4.6339 PMC10501234

[50] Wakeam E, Acuna SA, Leighl NB, et al. Surgery versus chemotherapy and radiotherapy for early and locally advanced small cell lung cancer: A Propensity-Matched analysis of survival. Lung Cancer. 2017;109:78–88. 10.1016/j.lungcan.2017.04.021. 28577955 10.1016/j.lungcan.2017.04.021

[51] Albayati A, Douedi S, Alshami A, et al. Why do patients leave against medical advice?? Reasons, consequences, prevention, and interventions. Healthc (Basel). 2021;9(2):111. 10.3390/healthcare9020111. 10.3390/healthcare9020111 PMC7909809 33494294

[52] Suh WN, Kong KA, Han Y, et al. Risk factors associated with treatment refusal in lung cancer. Thorac Cancer. 2017;8(5):443–50. 10.1111/1759-7714.12461. 28627788 10.1111/1759-7714.12461 PMC5582461

[53] Levy M, Buckell J, Clarke R, et al. Association between health insurance cost-sharing and choice of hospital tier for cardiovascular diseases in China: a prospective cohort study. The Lancet Reg Health– Western Pac. 2024;45. 10.1016/j.lanwpc.2024.101020. 10.1016/j.lanwpc.2024.101020 PMC10876671 38380231

[54] Sutherland K, Levesque J. Unwarranted clinical variation in health care: definitions and proposal of an analytic framework. J Eval Clin Pract. 2020;26(3):687–96. 10.1111/jep.13181. 31136047 10.1111/jep.13181 PMC7317701

[55] Meyers DS, Mishori R, McCann J, Delgado J, O’Malley AS, Fryer E. Primary care physicians’ perceptions of the effect of insurance status on clinical decision making. Ann Fam Med. 2006;4(5):399–402. 10.1370/afm.574. 17003138 10.1370/afm.574 PMC1578641

[56] Hu T, Decker SL, Chou SY. The impact of health insurance expansion on physician treatment choice: medicare part D and physician prescribing. Int J Health Econ Manag Published Online Febr 6, 2017:10.1007/s10754-017-9211-9212. 10.1007/s10754-017-9211-2 PMC6606398 28168448

[57] Clemens J, Gottlieb JD. Do physicians’ financial incentives affect medical treatment and patient health?? Am Econ Rev. 2014;104(4):1320–49. 10.1257/aer.104.4.1320. 25170174 10.1257/aer.104.4.1320 PMC4144420

[58] Li Y, Yang Y, Yuan J, Huang L, Ma Y, Shi X. Differences in medical costs among urban lung cancer patients with different health insurance schemes: a retrospective study. BMC Health Serv Res. 2022;22(1):612. 10.1186/s12913-022-07957-9. 35524258 10.1186/s12913-022-07957-9 PMC9077891

[59] Yang Y, Man X, Nicholas S, et al. Utilisation of health services among urban patients who had an ischaemic stroke with different health insurance - a cross-sectional study in China. BMJ Open. 2020;10(10):e040437. 10.1136/bmjopen-2020-040437. 33040017 10.1136/bmjopen-2020-040437 PMC7549448

[60] Mao W, Tang S, Zhu Y, Xie Z, Chen W. Financial burden of healthcare for cancer patients with social medical insurance: a multi-centered study in urban China. Int J Equity Health. 2017;16(1):180. 10.1186/s12939-017-0675-y. 29017542 10.1186/s12939-017-0675-y PMC5635570

[61] Gonzalez-Saenz de Tejada M, Bilbao A, Baré M, et al. Association between social support, functional status, and change in health-related quality of life and changes in anxiety and depression in colorectal cancer patients. Psychooncology. 2017;26(9):1263–9. 10.1002/pon.4303. 28872742 10.1002/pon.4303

[62] Nwagbara UI, Ginindza TG, Hlongwana KW. Lung cancer awareness and palliative care interventions implemented in low-and middle-income countries: a scoping review. BMC Public Health. 2020;20(1):1466. 10.1186/s12889-020-09561-0. 32993570 10.1186/s12889-020-09561-0 PMC7526234

