# Supplementary material for: Disparities in inpatient treatment and expenditures among lung cancer patients under tiered social health insurance: a population-based study in China
Source: Int J Equity Health. 2025 Jun 5;24:163. doi: 10.1186/s12939-025-02533-z (PMC12139372; doi:10.1186/s12939-025-02533-z)
Supplement: Supplementary file 1 — Supplementary Material 1 [file 12939_2025_2533_MOESM1_ESM.docx]

**Supplementary Content**

[Methodological appendix 2](#_Toc196303690)

[Part 1: Social health insurance schemes in China 2](#_Toc196303691)

[Part 2: Data source 3](#_Toc196303692)

[Part 3: Identification of new incidence cases and washout period 4](#_Toc196303693)

[Part 4: Main Regression Models 5](#_Toc196303694)

[Part 5: Propensity Score Matching 6](#_Toc196303695)

[Table S1 Medical codes used to identify lung cancer cases and specific-cause inpatient utilizations 7](#_Toc196303696)

[Table S2 Medical codes used to calculate Charlson Comorbidity Index at diagnosis 8](#_Toc196303697)

[Table S3 Medical codes used to identify lung cancer treatment 9](#_Toc196303698)

[Table S4 Descriptive Statistics for the Non-metastatic Group Sample, 2017-21 10](#_Toc196303699)

[Table S5 Descriptive Statistics for the Metastatic Group Sample, 2017-21 11](#_Toc196303700)

[Table S6 Descriptive Statistics for the Expenditure Sample, 2017-21 12](#_Toc196303701)

[References 14](#_Toc196303702)

# Methodological appendix

# Part 1: Social health insurance schemes in China

China’s Social Health Insurance (SHI) system has evolved significantly over the past few decades, achieving near-universal coverage of approximately 95% of the population by 2023. The system comprises two primary schemes: the Urban Employee Basic Medical Insurance (UEBMI) and the Urban and Rural Resident Basic Medical Insurance (URRBMI), which together cover the majority of SHI beneficiaries.

UEBMI was introduced in 1998 as part of China’s healthcare reforms to provide medical insurance for urban workers. URRBMI was established in 2016 through the merger of two earlier schemes: the Urban Resident Basic Medical Insurance and the New Rural Cooperative Medical Scheme. The Urban Resident Basic Medical Insurance, launched in 2007, targeted non-working urban residents, while the New Rural Cooperative Medical Scheme, introduced in 2003, focused on rural populations.

Table S1.1 Characteristics of China's two basic medical insurance systems

|  | UEBMI | URRBMI |
| --- | --- | --- |
| **Target Population** | Employees and retirees with formal employment | Urban non-employed individuals and rural residents |
| **Funding** | Employer (6% payroll) + employee (2% salary) | Gov. subsidy (≥RMB 610, 2022) + individual (RMB 350, 2022) |
| **Contribution Period** | 15–35 years for lifelong coverage | Pay-as-you-go |
| **Inpatient Benefits (Jinan, Shandong, 2024)** |  |  |
| **Deductible** |  |  |
| Tertiary Hospital | 1st: RMB 1,000; 2nd: RMB 500; 3rd+: RMB 0 | RMB 1,000 |
| Secondary Hospital | 1st: RMB 400; 2nd: RMB 200; 3rd+: RMB 0 | RMB 400 |
| Primary Hospital |  | RMB 400 |
| Community Hospital/Township Health Center | 1st: RMB 200; 2nd: RMB 100; 3rd+: RMB 0 | RMB 200 |
| **Reimbursement Rate** |  |  |
| Provincial/Ministerial Tertiary Hospital | Up to RMB 10,000: 85%;  RMB 10,000–400,000: 88%;  RMB 400,000–600,000: 90% | 50% |
| Other Tertiary Hospitals |  | 60% |
| Secondary Hospital | Up to RMB 10,000: 90%;  RMB 10,000–400,000: 93%;  RMB 400,000–600,000: 90% | 70% |
| Primary Hospital |  | 80% |
| Township Health Center |  | 90% |
| **Max Coverage** | 1st: RMB 1,000; 2nd: RMB 500; 3rd+: RMB 0 | RMB 250,000 (incl. individual share) |

# Part 2: Data source

The data for this study were sourced from the Cheeloo Lifespan Electronic Health Research Data-library (Cheeloo LEAD), an electronic health data platform developed by the National Health Medical Big Data Research Institute (Northern Center). The database adheres to the reporting standards of the National Health Direct Reporting System and the 2018 edition of the Shandong Provincial Population Health Information Platform Shared Dataset. It is updated daily, with data for a given day uploaded the following day. Adhering to the data reporting standards of the National Health Direct Reporting System and the Shandong Provincial Population Health Information Platform Shared Dataset (2018 edition), the database updates in a t+1 manner. In other words, the data for a given day would be uploaded on the following day. Further details regarding the platform's information are available at the official website (<http://www.mhdata.sdu.edu.cn/cheeloolead.htm>) and in prior scholarly publications^1–4^.

The dataset includes hospitalization record front pages (HRFPs) from all secondary and tertiary hospitals in Shandong since 2009, covering demographics, residence, hospitalization details, and other basic characteristics. Data on the platform were anonymized, with individual identity number replaced by a unique encrypted identity number.

# Part 3: Identification of new incidence cases and washout period

In this study, we used a washout period of at least 4 years to identify new incidence cases in 2017-2021, as the number of lung cancer patients in 2021 remained stable when the time window was reset from 4 years (2017-2020) to 8 years (2013-2020) (Figure 1). For example, we used a 4-year washout period to define cancer cases in individuals whose earliest admission event date of report card was between Jan 1, 2017, and Dec 31, 2017, as new incidence cases in 2017. Meanwhile, we defined a lung incident cancer case on the basis of the first definite lung cancer related HRFPs, taking the corresponding date of admission as the date of diagnosis.


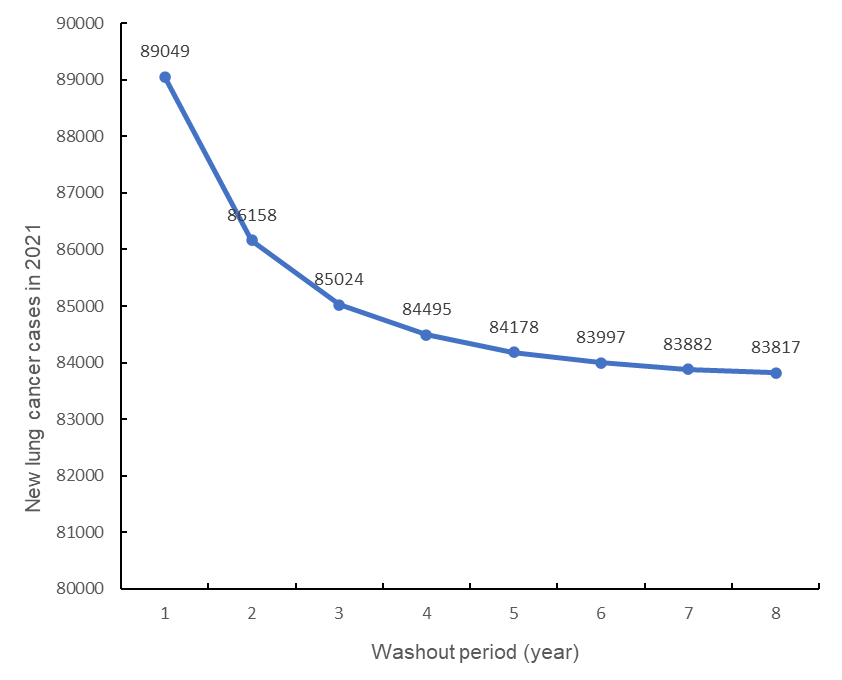


Figure S1 Number of new lung cancer cases in 2021, based on the washout time window from 1 to 8 years.

# Part 4: Main Regression Models

We utilized multivariable logistic regressions to measure associations between health insurance status and the receipt of treatment. The equations for the treatment pattern outcome models were specified as follows:

$${Treat}_{it}=\beta_{0}+\beta_{1}{InusranceType}_{it}+\beta_{2}X_{it}+\beta_{3}{NSCLC}_{it}+\beta_{4}{Comorbidities}_{it}+\beta_{5}\mathrm{HospitalLevel}_{it}+\beta_{6}{\mathrm{HospitalR}egion}_{it}+\mu{Year}_{t}+\varepsilon_{i}$$

where *Treat* is the treatment pattern, *i* indexes the individual and *t* the year. $X_{it}$ is a vector of demographics (sex, age group, marry, ethnicity, and occupation), *Comorbidities* and *NSCLC*. *μ* is a vector of year fixed effects (for 2017 - 2021). The coefficient of interest is $\beta_{1}$, which measures the outcome difference, making that person eligible for UEBMI coverage, rather than URRBMI.

All coefficients were then converted into adjusted outcome estimates using the “margins” command in R for UEBMI-Eligible, which provides separate sample-wide marginal outcomes for the UEBMI-Eligible population and the URRBMI-Eligible population, using each observations’ actual covariates and the coefficients from the relevant regression model described above^5^.

Medical expenditures data are characterized by pronounced positive skewness, with a long right tail driven by a minority of high-cost cases. In case of the absence of censoring and zero-cost observations, a vast body of literature supports the application of Generalized Linear Models (GLMs) with a Gamma distribution and log link function for modeling medical expenditures^7^. Empirical evidence suggests that GLMs, especially the Gamma regression model, behaved well in estimating population means of medical expenditures ^6^.

Building upon this, our regression analysis used GLMs, using the distributions and link functions described in the main methods of the paper^7^. The equations for the expenditure outcome models were specified as follows:

$${E\mathrm{xpenditure}}_{it}=\beta_{0}+\beta_{1}{InusranceType}_{it}+\beta_{2}X_{it}+\beta_{3}{NSCLC}_{it}+\beta_{4}{Comorbidities}_{it}+\beta_{5}\mathrm{HospitalLevel}_{it}+\beta_{6}{\mathrm{HospitalR}egion}_{it}+\mu{Year}_{t}+\varepsilon_{i}$$

where *Expenditures* is the medical expenditures for lung cancer patients.

# Part 5: Propensity Score Matching

To minimize confounding in our analysis of outcome indicators between Urban Employee Basic Medical Insurance (UEBMI) and Urban and Rural Resident Insurance (URRBMI) patients, we implemented propensity score matching (PSM)^8^. Our propensity score model was based on a logistic regression of the following form:

$$Logit\left( {InsuranceType}_{it} \right)=\beta_{0}+\beta_{1}{Age}_{it}+\beta_{2}{Gender}_{it}+\beta_{3}{Ethnicity}_{it}+\beta_{4}{Marry}_{it}+\beta_{5}\mathrm{Occupation}_{it}+\beta_{6}{Year}_{it}+\varepsilon_{i}$$

In this model, the dependent variable, *InsuranceType*, indicates the type of insurance (0 for URRBMI and 1 for UEBMI), while *Age,* *Gender*, *Ethnicity, Marry,* *Occupation, and Year* represent individual-level covariates. We performed matching using a 1:1 nearest neighbor approach with a caliper of 0.2 of the standard deviation. After matching, we assessed the balance of covariates using standardized mean differences (SMD), with a threshold of ≤ 0.1 indicating adequate balance. This process allowed us to create a balanced sample for subsequent regression analyses.

# Table S1 Medical codes used to identify lung cancer cases and specific-cause inpatient utilizations

|  | ICD-10 Codes |
| --- | --- |
| Lung cancer cases | C34 |
| Specific-cause inpatient utilizations | Z51, C34, Z08, Z54, C79, C78, C77 |

# Table S2 Medical codes used to calculate Charlson Comorbidity Index at diagnosis

| Score | ICD-10 Codes |
| --- | --- |
| Score =1 | "I21", "I22", "I25.2", "I09.9", "I11.0", "I13.0", "I13.2", "I25.5", "I42.0", "I42.5", "I42.6", "I42.7", "I42.8", "I42.9", "I43", "I50", "P29.0", "I70", "I71", "I73.1", "I73.8", "I73.9", "I77.1", "I79.0", "I79.2", "K55.1", "K55.8", "K55.9", "Z95.8", "Z95.9", "G45", "G46", "I60", "I61", "I62", "I63", "I64", "I65", "I66", "I67", "I68", "I69", "H34.0", "F00", "F01", "F02", "F03", "G30", "F051", "G31.1", "I27.8", "I27.9", "J40", "J41", "J42", "J43", "J44", "J45", "J46", "J47", "J60", "J61", "J62", "J63", "J64", "J65", "J66", "J67", "J68.4", "J70.1", "J70.3", "M05", "M06", "M31.5", "M32", "M33", "M34", "M35.1", "M35.3", "M36.0", "K25", "K26", "K27", "K28", "B18", "K70.0", "K70.1", "K70.2", "K70.3", "K70.9", "K71.3", "K71.4", "K71.5", "K71.7", "K73", "K74", "K76.0", "K76.2", "K62.3", "K76.4", "K76.8", "K76.9", "Z94.4", "E10.0", "E10.1", "E10.6", "E10.8", "E10.9", "E11.0", "E11.1", "E11.6", "E11.8", "E11.9", "E12.0", "E12.1", "E12.6", "E12.8", "E12.9", "E13.0", "E13.1", "E13.6", "E13.8", "E13.9", "E14.0", "E14.1", "E14.6", "E14.8", "E14.9" |
| Score =2 | "G04.1", "G11.4", "G80.1", "G80.2", "G81", "G82", "G83.0", "G83.1", "G83.2", "G83.3", "G83.4", "G83.9", "I12.0", "I13.1", "N03.2", "N03.3", "N03.4", "N03.5", "N03.6", "N03.7", "N05.2", "N05.3", "N05.4", "N05.5", "N05.6", "N05.7", "N18", "N19", "N25.0", "Z49.0", "Z49.1", "Z49.2", "Z94.0", "Z99.2", "E10.2", "E10.3", "E10.4", "E10.5", "E10.7", "E11.2", "E11.5", "E11.7", "E12.2", "E12.3", "E12.4", "E12.5", "E12.7", "E13.2", "E13.3", "E13.4", "E13.5", "E13.7", "E14.2", "E14.3", "E14.4", "E14.5", "E14.7" |
| Score =3 | "I85.9", "I86.4", "I98.2", "K70.4", "K71.1", "K72.1", "K72.9", "K76.5", "K76.7", "K76.9" |
| Score =6 | "B20", "B21", "B22", "B24" |

# Table S3 Medical codes used to identify lung cancer treatment

| Treatment | Coding System | Codes |
| --- | --- | --- |
| Surgery | ICD-10 | Z54.0, Z54.000, Z54.001 |
|  | ICD-9-CM3 | 31.5, 31.5x00, 31.5x00x003, 31.5x00x012, 31.5x00x013, 31.5x00x014, 31.5x00x015, 31.5x01, 31.5x02, 31.5x03, 31.5x04, 32.0, 32.01, 32.09, 32.1, 32.1x00, 32.1x00x004, 32.1x01, 32.1x02, 32.1x03, 32.1x04, 32.2, 32.20, 32.23, 32.24, 32.25, 32.26, 32.28, 32.29, 32.3, 32.30, 32.39, 32.4, 32.41, 32.49, 32.5, 32.50, 32.59, 32.6, 32.6x00, 32.6x00x002, 32.6x00x004, 32.9, 32.9x00 |
| Chemotherapy | ICD-10 | Z51.1, Z51.100, Z51.101, Z51.102, Z51.103, Z51.104, Z51.2, Z51.200, Z54.2, Z54.200 |
|  | ICD-9-CM3 | 99.25, 99.2500, 99.2500x017, 99.2500x037, 99.2500x038, 99.2500x039, 99.2501, 99.2502, 99.2503, 99.2504, 99.2505, 99.2506 |
| Radiotherapy | ICD-10 | Z51.0, Z51.000, Z51.001, Z51.002, Z51.003, Z54.1, Z54.100 |
|  | ICD-9-CM3 | 92.2, 92.20, 92.21, 92.22, 92.23, 92.24, 92.25, 92.26, 92.27, 92.28, 92.29, 92.41 |
| Targeted Therapy | ICD-10 | Z51.801, Z51.807 |
|  | ICD-9-CM3 | 99.2800x006 |
| Immunotherapy | ICD-10 | Z51.805, Z51.810 |
|  | ICD-9-CM3 | 99.2801, 99.2800x004, 99.2800x005 |

# Table S4 Descriptive Statistics for the Non-metastatic Group Sample, 2017-21

|  | **Full sample** | | |  | **Propensity score–matched sample** | | |
| --- | --- | --- | --- | --- | --- | --- | --- |
|  | **No. (%)** |  |  |  | **No. (%)** |  |  |
|  | URRBMI | UEBMI |  |  | URRBMI | UEBMI |  |
|  | (N=146405) | (N=80374) | SMD |  | (N=47170) | (N=47170) | SMD |
| **Matching Variables** |  |  |  |  |  |  |  |
| Year of diagnosis |  |  |  |  |  |  |  |
| 2017 | 22753 (15.5%) | 11599 (14.4%) | -0.0111 |  | 7444 (15.8%) | 6518 (13.8%) | -0.0196 |
| 2018 | 25579 (17.5%) | 13787 (17.2%) | -0.0032 |  | 8579 (18.2%) | 8160 (17.3%) | -0.0089 |
| 2019 | 29085 (19.9%) | 16537 (20.6%) | 0.0071 |  | 9793 (20.8%) | 10717 (22.7%) | 0.0196 |
| 2020 | 32032 (21.9%) | 17953 (22.3%) | 0.0046 |  | 10795 (22.9%) | 10194 (21.6%) | -0.0127 |
| 2021 | 36956 (25.2%) | 20498 (25.5%) | 0.0026 |  | 10559 (22.4%) | 11581 (24.6%) | 0.0217 |
| Age at diagnosis |  |  |  |  |  |  |  |
| Mean (SD), years | 65.3 (10.1) | 63.0 (11.5) |  |  | 63.5 (10.7) | 62.2 (11.1) |  |
| <45 | 3363 (2.3%) | 4722 (5.9%) | 0.0358 |  | 1848 (3.9%) | 1999 (4.2%) | 0.0032 |
| 45-59 | 36174 (24.7%) | 24561 (30.6%) | 0.0585 |  | 14679 (31.1%) | 18366 (38.9%) | 0.0782 |
| 60-75 | 85189 (58.2%) | 39662 (49.3%) | -0.0884 |  | 24344 (51.6%) | 20861 (44.2%) | -0.0738 |
| >75 | 21679 (14.8%) | 11429 (14.2%) | -0.0059 |  | 6299 (13.4%) | 5944 (12.6%) | -0.0075 |
| Gender |  |  |  |  |  |  |  |
| Male | 86139 (58.8%) | 49423 (61.5%) |  |  | 28434 (60.3%) | 26798 (56.8%) |  |
| Female | 60266 (41.2%) | 30951 (38.5%) | -0.0266 |  | 18736 (39.7%) | 20372 (43.2%) | 0.0347 |
| Ethnicity |  |  |  |  |  |  |  |
| Han | 144902 (99.0%) | 79637 (99.1%) |  |  | 46741 (99.1%) | 46682 (99.0%) |  |
| Other | 1503 (1.0%) | 737 (0.9%) | -0.0011 |  | 429 (0.9%) | 488 (1.0%) | 0.0013 |
| Marital status |  |  |  |  |  |  |  |
| Single | 3439 (2.3%) | 1149 (1.4%) | -0.0092 |  | 477 (1.0%) | 939 (2.0%) | 0.0098 |
| Married | 139681 (95.4%) | 77860 (96.9%) | 0.0146 |  | 46100 (97.7%) | 45467 (96.4%) | -0.0134 |
| Divorced | 3285 (2.2%) | 1365 (1.7%) | -0.0055 |  | 593 (1.3%) | 764 (1.6%) | 0.0036 |
| Occupation |  |  |  |  |  |  |  |
| Employees/workers | 5773 (3.9%) | 17307 (21.5%) | 0.1759 |  | 5773 (12.2%) | 5252 (11.1%) | -0.011 |
| Non-practitioners | 97879 (66.9%) | 12456 (15.5%) | -0.5136 |  | 12456 (26.4%) | 12456 (26.4%) | 0.0000 |
| Special Employees | 4173 (2.9%) | 25196 (31.3%) | 0.285 |  | 4173 (8.8%) | 5311 (11.3%) | 0.0241 |
| Unspecified | 38580 (26.4%) | 25415 (31.6%) | 0.0527 |  | 24768 (52.5%) | 24151 (51.2%) | -0.0131 |
| **Non-Matching Variables** |  |  |  |  |  |  |  |
| Type of lung cancer |  |  |  |  |  |  |  |
| SCLC | 15071 (10.3%) | 5367 (6.7%) |  |  | 4620 (9.8%) | 3200 (6.8%) |  |
| NSCLC | 88598 (60.5%) | 57931 (72.1%) |  |  | 30649 (65.0%) | 33853 (71.8%) |  |
| Unspecified | 42736 (29.2%) | 17076 (21.2%) |  |  | 11901 (25.2%) | 10117 (21.4%) |  |
| CCI |  |  |  |  |  |  |  |
| CCI = 0 | 89482 (61.1%) | 49704 (61.8%) |  |  | 29598 (62.7%) | 29742 (63.1%) |  |
| CCI = 1 | 37708 (25.8%) | 20287 (25.2%) |  |  | 11840 (25.1%) | 11687 (24.8%) |  |
| CCI >= 2 | 19215 (13.1%) | 10383 (12.9%) |  |  | 5732 (12.2%) | 5741 (12.2%) |  |
| Hospital level |  |  |  |  |  |  |  |
| Secondary hospitals | 45652 (31.2%) | 13049 (16.2%) |  |  | 10757 (22.8%) | 7791 (16.5%) |  |
| Tertiary hospitals | 99752 (68.1%) | 66932 (83.3%) |  |  | 36111 (76.6%) | 39136 (83.0%) |  |
| Unclassified or Other | 1001 (0.7%) | 393 (0.5%) |  |  | 302 (0.6%) | 243 (0.5%) |  |
| Hospital region |  |  |  |  |  |  |  |
| Eastern (Peninsula) Region | 25177 (17.2%) | 27554 (34.3%) |  |  | 14855 (31.5%) | 18351 (38.9%) |  |
| Northern Region | 22388 (15.3%) | 7117 (8.9%) |  |  | 4887 (10.4%) | 3710 (7.9%) |  |
| Southern Region | 46676 (31.9%) | 10586 (13.2%) |  |  | 10534 (22.3%) | 6518 (13.8%) |  |
| Central Region | 52164 (35.6%) | 35117 (43.7%) |  |  | 16894 (35.8%) | 18591 (39.4%) |  |

# Table S5 Descriptive Statistics for the Metastatic Group Sample, 2017-21

|  | **Full sample** | | |  | **Propensity score–matched sample** | | |
| --- | --- | --- | --- | --- | --- | --- | --- |
|  | **No. (%)** |  |  |  | **No. (%)** |  |  |
|  | URRBMI | UEBMI |  |  | URRBMI | UEBMI |  |
|  | (N=46279) | (N=17265) | SMD |  | (N=11298) | (N=11298) | SMD |
| **Matching Variables** |  |  |  |  |  |  |  |
| Year of diagnosis |  |  |  |  |  |  |  |
| 2017 | 5824 (12.6%) | 2648 (15.3%) | 0.0275 |  | 1897 (16.8%) | 2360 (20.9%) | 0.041 |
| 2018 | 7327 (15.8%) | 2929 (17.0%) | 0.0113 |  | 2095 (18.5%) | 1810 (16.0%) | -0.0252 |
| 2019 | 9062 (19.6%) | 3522 (20.4%) | 0.0082 |  | 2255 (20.0%) | 2491 (22.0%) | 0.0209 |
| 2020 | 10941 (23.6%) | 3931 (22.8%) | -0.0087 |  | 2498 (22.1%) | 2255 (20.0%) | -0.0215 |
| 2021 | 13125 (28.4%) | 4235 (24.5%) | -0.0383 |  | 2553 (22.6%) | 2382 (21.1%) | -0.0151 |
| Age at diagnosis |  |  |  |  |  |  |  |
| Mean (SD), years | 66.2 (10.2) | 65.6 (11.6) |  |  | 64.7 (11.2) | 64.5 (11.7) |  |
| <45 | 999 (2.2%) | 696 (4.0%) | 0.0187 |  | 474 (4.2%) | 522 (4.6%) | 0.0042 |
| 45-59 | 10389 (22.4%) | 4226 (24.5%) | 0.0203 |  | 3095 (27.4%) | 3200 (28.3%) | 0.0093 |
| 60-75 | 26731 (57.8%) | 8762 (50.8%) | -0.0701 |  | 5737 (50.8%) | 5505 (48.7%) | -0.0205 |
| >75 | 8160 (17.6%) | 3581 (20.7%) | 0.0311 |  | 1992 (17.6%) | 2071 (18.3%) | 0.007 |
| Gender |  |  |  |  |  |  |  |
| Male | 27048 (58.4%) | 11960 (69.3%) |  |  | 7403 (65.5%) | 7661 (67.8%) |  |
| Female | 19231 (41.6%) | 5305 (30.7%) | -0.1083 |  | 3895 (34.5%) | 3637 (32.2%) | -0.0228 |
| Ethnicity |  |  |  |  |  |  |  |
| Han | 45732 (98.8%) | 17088 (99.0%) |  |  | 11201 (99.1%) | 11187 (99.0%) |  |
| Other | 547 (1.2%) | 177 (1.0%) | -0.0016 |  | 97 (0.9%) | 111 (1.0%) | 0.0012 |
| Marital status |  |  |  |  |  |  |  |
| Single | 1546 (3.3%) | 320 (1.9%) | -0.0149 |  | 148 (1.3%) | 195 (1.7%) | 0.0042 |
| Married | 43358 (93.7%) | 16468 (95.4%) | 0.017 |  | 10917 (96.6%) | 10811 (95.7%) | -0.0094 |
| Divorced | 1375 (3.0%) | 477 (2.8%) | -0.0021 |  | 233 (2.1%) | 292 (2.6%) | 0.0052 |
| Occupation |  |  |  |  |  |  |  |
| Employees/workers | 1519 (3.3%) | 2917 (16.9%) | 0.1361 |  | 1519 (13.4%) | 1502 (13.3%) | -0.0015 |
| Non-practitioners | 32277 (69.7%) | 3589 (20.8%) | -0.4896 |  | 3588 (31.8%) | 3589 (31.8%) | 0.0001 |
| Special Employees | 957 (2.1%) | 5637 (32.6%) | 0.3058 |  | 957 (8.5%) | 1085 (9.6%) | 0.0113 |
| Unspecified | 11526 (24.9%) | 5122 (29.7%) | 0.0476 |  | 5234 (46.3%) | 5122 (45.3%) | -0.0099 |
| **Non-Matching Variables** |  |  |  |  |  |  |  |
| Type of lung cancer |  |  |  |  |  |  |  |
| SCLC | 5580 (12.1%) | 2072 (12.0%) |  |  | 1396 (12.4%) | 1313 (11.6%) |  |
| NSCLC | 26879 (58.1%) | 10742 (62.2%) |  |  | 6700 (59.3%) | 7001 (62.0%) |  |
| Unspecified | 13820 (29.9%) | 4451 (25.8%) |  |  | 3202 (28.3%) | 2984 (26.4%) |  |
| CCI |  |  |  |  |  |  |  |
| CCI = 0 | 29778 (64.3%) | 10904 (63.2%) |  |  | 7354 (65.1%) | 7274 (64.4%) |  |
| CCI = 1 | 12054 (26.0%) | 4495 (26.0%) |  |  | 2892 (25.6%) | 2861 (25.3%) |  |
| CCI >= 2 | 4447 (9.6%) | 1866 (10.8%) |  |  | 1052 (9.3%) | 1163 (10.3%) |  |
| Hospital level |  |  |  |  |  |  |  |
| Secondary hospitals | 13570 (29.3%) | 2791 (16.2%) |  |  | 2464 (21.8%) | 1755 (15.5%) |  |
| Tertiary hospitals | 32566 (70.4%) | 14418 (83.5%) |  |  | 8801 (77.9%) | 9503 (84.1%) |  |
| Unclassified or Other | 143 (0.3%) | 56 (0.3%) |  |  | 33 (0.3%) | 40 (0.4%) |  |
| Hospital region |  |  |  |  |  |  |  |
| Eastern (Peninsula) Region | 7338 (15.9%) | 5211 (30.2%) |  |  | 3209 (28.4%) | 3804 (33.7%) |  |
| Northern Region | 8106 (17.5%) | 1644 (9.5%) |  |  | 1327 (11.7%) | 958 (8.5%) |  |
| Southern Region | 14128 (30.5%) | 2283 (13.2%) |  |  | 2361 (20.9%) | 1528 (13.5%) |  |
| Central Region | 16707 (36.1%) | 8127 (47.1%) |  |  | 4401 (39.0%) | 5008 (44.3%) |  |

# Table S6 Descriptive Statistics for the Expenditure Sample, 2017-21

|  | **Full sample** | | |  | **Propensity score–matched sample** | | |
| --- | --- | --- | --- | --- | --- | --- | --- |
|  | **No. (%)** |  |  |  | **No. (%)** |  |  |
|  | **URRBMI** | **UEBMI** |  |  | **URRBMI** | **UEBMI** |  |
|  | **(N=138551)** | **(N=70484)** | **SMD** |  | **(N=40820)** | **(N=40820)** | **SMD** |
| **Matching variables** |  |  |  |  |  |  |  |
| Year of diagnosis |  |  |  |  |  |  |  |
| 2017 | 12094 (8.7%) | 6172 (8.8%) | 0.0003 |  | 3801 (9.3%) | 5010 (12.3%) | 0.0296 |
| 2018 | 14755 (10.6%) | 7765 (11.0%) | 0.0037 |  | 4651 (11.4%) | 4100 (10.0%) | -0.0135 |
| 2019 | 21496 (15.5%) | 11498 (16.3%) | 0.008 |  | 6885 (16.9%) | 6285 (15.4%) | -0.0147 |
| 2020 | 40314 (29.1%) | 20575 (29.2%) | 0.0009 |  | 12483 (30.6%) | 12536 (30.7%) | 0.0013 |
| 2021 | 49892 (36.0%) | 24474 (34.7%) | -0.0129 |  | 13000 (31.8%) | 12889 (31.6%) | -0.0027 |
| Age at diagnosis, years |  |  |  |  |  |  |  |
| Mean (SD) | 65.6 (10.1) | 63.1 (11.7) |  |  | 63.6 (10.8) | 62.3 (11.3) |  |
| Age group |  |  |  |  |  |  |  |
| <45 | 3180 (2.3%) | 4260 (6.0%) | 0.0375 |  | 1715 (4.2%) | 1997 (4.9%) | 0.0069 |
| 45-59 | 33643 (24.3%) | 21514 (30.5%) | 0.0624 |  | 12646 (31.0%) | 15353 (37.6%) | 0.0663 |
| 60-75 | 80231 (57.9%) | 34314 (48.7%) | -0.0922 |  | 20781 (50.9%) | 18309 (44.9%) | -0.0606 |
| >75 | 21497 (15.5%) | 10396 (14.7%) | -0.0077 |  | 5678 (13.9%) | 5161 (12.6%) | -0.0127 |
| Gender |  |  |  |  |  |  |  |
| Male | 79686 (57.5%) | 42870 (60.8%) |  |  | 24225 (59.3%) | 23523 (57.6%) |  |
| Female | 58865 (42.5%) | 27614 (39.2%) | -0.0331 |  | 16595 (40.7%) | 17297 (42.4%) | 0.0172 |
| Ethnicity |  |  |  |  |  |  |  |
| Han | 137066 (98.9%) | 69828 (99.1%) |  |  | 40484 (99.2%) | 40344 (98.8%) |  |
| Other | 1485 (1.1%) | 656 (0.9%) | -0.0014 |  | 336 (0.8%) | 476 (1.2%) | 0.0034 |
| Marital status at diagnosis |  |  |  |  |  |  |  |
| Single | 3026 (2.2%) | 946 (1.3%) | -0.0084 |  | 425 (1.0%) | 556 (1.4%) | 0.0032 |
| Married | 131810 (95.1%) | 68127 (96.7%) | 0.0152 |  | 39764 (97.4%) | 39362 (96.4%) | -0.0098 |
| Divorced/widowed/separated | 3715 (2.7%) | 1411 (2.0%) | -0.0068 |  | 631 (1.5%) | 902 (2.2%) | 0.0066 |
| Occupation |  |  |  |  |  |  |  |
| Employees/workers | 5142 (3.7%) | 15657 (22.2%) | 0.185 |  | 5142 (12.6%) | 4250 (10.4%) | -0.0219 |
| Non-practitioners | 94080 (67.9%) | 11033 (15.7%) | -0.5225 |  | 11033 (27.0%) | 11033 (27.0%) | 0 |
| Special Employees | 4109 (3.0%) | 22765 (32.3%) | 0.2933 |  | 4109 (10.1%) | 5343 (13.1%) | 0.0302 |
| Other or unspecified | 35220 (25.4%) | 21029 (29.8%) | 0.0441 |  | 20536 (50.3%) | 20194 (49.5%) | -0.0084 |
| **Non-matching variables** |  |  |  |  |  |  |  |
| Type of Lung Cancer |  |  |  |  |  |  |  |
| SCLC | 14483 (10.5%) | 4879 (6.9%) |  |  | 4086 (10.0%) | 2855 (7.0%) |  |
| NSCLC | 87185 (62.9%) | 52029 (73.8%) |  |  | 27159 (66.5%) | 29782 (73.0%) |  |
| Unclassified or Other | 36883 (26.6%) | 13576 (19.3%) |  |  | 9575 (23.5%) | 8183 (20.0%) |  |
| Tumour metastasis |  |  |  |  |  |  |  |
| No | 103922 (75.0%) | 58491 (83.0%) |  |  | 31536 (77.3%) | 33724 (82.6%) |  |
| Yes | 34629 (25.0%) | 11993 (17.0%) |  |  | 9284 (22.7%) | 7096 (17.4%) |  |
| CCI |  |  |  |  |  |  |  |
| CCI = 0 | 83365 (60.2%) | 43513 (61.7%) |  |  | 25325 (62.0%) | 25568 (62.6%) |  |
| CCI = 1 | 37074 (26.8%) | 18078 (25.6%) |  |  | 10502 (25.7%) | 10294 (25.2%) |  |
| CCI >= 2 | 18112 (13.1%) | 8893 (12.6%) |  |  | 4993 (12.2%) | 4958 (12.1%) |  |
| Hospital level |  |  |  |  |  |  |  |
| Secondary hospitals | 40787 (29.4%) | 10623 (15.1%) |  |  | 9016 (22.1%) | 5951 (14.6%) |  |
| Tertiary hospitals | 97005 (70.0%) | 59564 (84.5%) |  |  | 31572 (77.3%) | 34676 (84.9%) |  |
| Unclassified or Other | 759 (0.5%) | 297 (0.4%) |  |  | 232 (0.6%) | 193 (0.5%) |  |
| Hospital region |  |  |  |  |  |  |  |
| Eastern (Peninsula) Region | 23654 (17.1%) | 22741 (32.3%) |  |  | 12527 (30.7%) | 14358 (35.2%) |  |
| Northern Region | 22913 (16.5%) | 6484 (9.2%) |  |  | 4232 (10.4%) | 3156 (7.7%) |  |
| Southern Region | 41630 (30.0%) | 9045 (12.8%) |  |  | 8363 (20.5%) | 5680 (13.9%) |  |
| Central Region | 50354 (36.3%) | 32214 (45.7%) |  |  | 15698 (38.5%) | 17626 (43.2%) |  |

# References

1. Zhang Y, Hu M, Xiang B, Yu H, Wang Q. Urban–rural disparities in the association of nitrogen dioxide exposure with cardiovascular disease risk in China: effect size and economic burden. *International Journal for Equity in Health*. 2024;23(1):22. doi:10.1186/s12939-024-02117-3

2. Zhao L, Wang HT, Ye RZ, et al. Profile and dynamics of infectious diseases: a population-based observational study using multi-source big data. *BMC Infect Dis*. 2022;22(1):332. doi:10.1186/s12879-022-07313-6

3. Du WY, Yin CN, Wang HT, et al. Infectious diseases among elderly persons: Results from a population-based observational study in Shandong province, China, 2013-2017. *J Glob Health*. 2021;11:08010. doi:10.7189/jogh.11.08010

4. Incidence trend and disease burden of seven vaccine-preventable diseases in Shandong province, China, 2013–2017: Findings from a population-based observational study. *Vaccine: X*. 2022;10:100145. doi:10.1016/j.jvacx.2022.100145

5. Norton EC, Dowd BE, Maciejewski ML. Marginal Effects-Quantifying the Effect of Changes in Risk Factors in Logistic Regression Models. *JAMA*. 2019;321(13):1304-1305. doi:10.1001/jama.2019.1954

6. Malehi AS, Pourmotahari F, Angali KA. Statistical models for the analysis of skewed healthcare cost data: a simulation study. *Health Econ Rev*. 2015;5:11. doi:10.1186/s13561-015-0045-7

7. Gregori D, Petrinco M, Bo S, Desideri A, Merletti F, Pagano E. Regression models for analyzing costs and their determinants in health care: an introductory review. *International Journal for Quality in Health Care*. 2011;23(3):331-341. doi:10.1093/intqhc/mzr010

8. Allen H, Gordon SH, Lee D, Bhanja A, Sommers BD. Comparison of Utilization, Costs, and Quality of Medicaid vs Subsidized Private Health Insurance for Low-Income Adults. *JAMA Netw Open*. 2021;4(1):e2032669. doi:10.1001/jamanetworkopen.2020.32669
